# Supplementary material for: Life beyond Fritz: On the Detachment of Electrolytic Bubbles
Source: Langmuir. 2024 Sep 21;40(39):20474–84. doi: 10.1021/acs.langmuir.4c01963 (PMC11447920; doi:10.1021/acs.langmuir.4c01963)
Supplement: Supplementary file 1 — la4c01963_si_003.pdf [file la4c01963_si_003.pdf]

Supporting Information: Life beyond Fritz: On the detachment of electrolytic bubbles

Çayan Demirkır,† Jeffery A. Wood,‡ Detlef Lohse,†,¶ and Dominik Krug*,†

†*Physics of Fluids, University of Twente, Enschede 7500 AE, The Netherlands*

‡*Soft Matter, Fluidics, and Interfaces, University of Twente, Enschede 7500 AE, The Netherlands*

¶*Max Planck Institute for Dynamics and Self-Organization, Am Fassberg 17, 37077 Göttingen, Germany*

E-mail: d.j.krug@utwente.nl

Contents

S1 Force Balance on a Bubble **2**

S2 Electrolyte Properties **4**

S3 Surface Characterization **5**

S4 Wetting Characteristics **8**

S5 Spreading Bubbles at Various Conditions **10**

References **10**

S1 Force Balance on a Bubble

In deriving the force balance, we restrict ourselves to the effects of surface tension, pressure and buoyancy. Other factors, such as thermal[1,2] or solutocapillary[3,4] Marangoni are also known to play a role, but are likely to be less relevant in the present case due to the low current densities in the experiment. The bubble growth is slow and occurs in the absence of external flow, such that inertial or other hydrodynamic forces are negligible. Note that we also neglect a possible contribution of an electric force, mainly because the wide range of reported surface charge density estimates in the literature[1,5] does not allow for a definitive evaluation of this contribution.

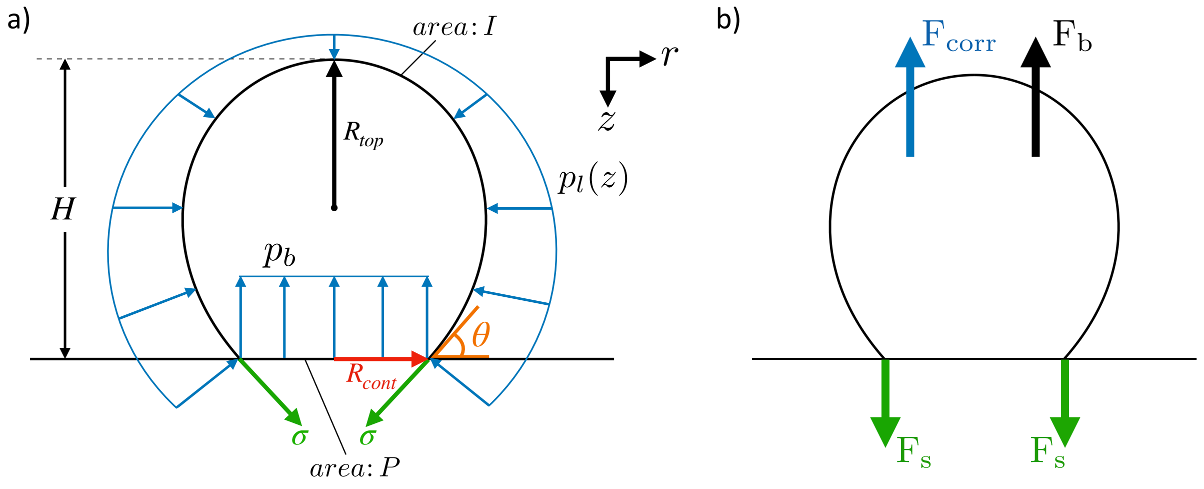

Figure S1: Schematics of the (a) stresses and (b) main forces acting on a bubble during growth.

We consider a bubble in a fluid at rest for which the hydrostatic pressure distribution is given by

$$p_l(z) = p_l(z = 0) + \rho_l g z, \tag{S1}$$

where ρ_l, g, and z denote liquid density, gravitational acceleration, and the vertical distance from the origin line at the bubble top ($z = 0$), respectively (See Figure S1a). Applying Laplace's law at the top of the bubble, the pressure p_b inside the bubble can be obtained from

$$p_b - p_l(z=0) = \frac{2\sigma}{R_{top}}, \tag{S2}$$

with σ denoting the surface tension and R_{top} the radius of curvature at the top of the bubble. Note that since $\rho_g \ll \rho_l$, p_b can be assumed to be constant within the bubble.

The resulting force in the vertical ($\vec{e_z}$) direction F_z on the bubble follows from the integration

$$F_z = -\iint_I p_l \vec{e_z} \cdot \vec{n} dA - \iint_P p_b \vec{e_z} \cdot \vec{n} dA + \oint_C \sigma \sin\theta dl. \tag{S3}$$

Here, "I" and "P" denote the surfaces (with the normal vector $\vec{n}$ pointing outward) of the gas-liquid interface and the contact patch, respectively, and "C" is the contact line. Noting that the buoyancy force $F_b = -\iint_{I+P} p_l(z)\vec{e_z} \cdot \vec{n} dA$ and using the surface tension force $F_s = \oint_C \sigma \sin\theta dl = 2\pi R_{cont}\sigma \sin\theta$, we obtain

$$F_z = -F_b - \iint_P (p_b - p_l(z))\vec{e_z} \cdot \vec{n} dA + F_s \tag{S4}$$

and

$$F_z = -F_b - \pi R_{cont}^2 (p_b - p_l(H)) + F_s. \tag{S5}$$

The forces with negative sign in equation S5 point upward (negative z direction), whereas surface tension force F_s acts downwards (positive z direction). Using equation (S2), the second term in the previous equation known as the 'pressure correction force' F_{corr} can be written as

$$F_{corr} = -\pi R_{cont}^2 \left(\frac{2\sigma}{R_{top}} - \rho_l g H \right). \tag{S6}$$

The correction force results from the fact that the expression for F_b assumes hydrostatic pressure at the bubble foot area P, whereas the actual pressure acting there is p_b. The first term in equation (S6) corresponds to the contact pressure force on the bubble foot while the second one represents a buoyancy correction. For a bubble to remain stationary, the

resulting vertical force must be zero *at all times*, which leads to the condition

$$\frac{dv}{dt} = F_z = 0 = F_b + F_{corr} + F_s \tag{S7}$$

S2 Electrolyte Properties

The electrolyte solutions used in the electrolysis experiments were prepared in different acid concentrations (from 10^{-4} M to 1 M). Furthermore, 0.5 M $NaClO_4 \cdot H_2O$ was added into the solutions as supporting electrolyte (except 1 M $HClO_4$ case). In this way, the electrical conductivity of the solution was increased and high ohmic overpotentials were avoided during electrolysis, so that experiments up to a current density of -50 A/m^2 could be performed for low acid concentrations. In this study, the bubble profile and contact angle were determined using the Young-Laplace (YL) equations, necessitating the calculation of the Bond number $\mathrm{Bo} = \Delta\rho \mathrm{g} \mathrm{R}_{\mathrm{top}}^2/\sigma$. Therefore, physical properties of the electrolytes such as density and surface tension should be characterized.

The density of the mixture solution was found by dividing the total mass of the solution by the total volume. The total mass and volume were calculated by summing the mass and volume of each substance, namely water, $HClO_4$ and $NaClO_4 \cdot H_2O$. On the other hand, the surface tension of the electrolytes was found by pendant drop measurements. The experiments were carried out with an optical contact angle goniometer (OCA 15 Pro from Dataphysics Instruments), and instrument's software (SCA20) was used to determine the drop shape and calculate the surface tension. Using a Hamilton syringe, a drop was generated on the tip of a needle (outer diameter of 0.718 mm) and pumped in very slowly (0.05 µL/s) until it starts swing up and down. Subsequently, the corresponding surface tension value is found from the shape of the drop at that moment. Here, selecting a sufficiently large needle is crucial to enhance the accuracy of the measurements, as it ensures the formation of a large drop with significant deformation. The measurements were repeated with five drops for each

liquids, and the average value were taken as σ value. The values of ρ and σ are shown in Table S1.

Table S1: Density (ρ) and surface tension (σ) of the electrolytes used in the experiments at 20°C

	ρ [kg/m^3]	σ [mN/m]
Water	1000	72.70 ± 0.09
10^{-4} M $HClO_4$ + 0.5 M $NaClO_4$	1035	70.23 ± 0.09
10^{-3} M $HClO_4$ + 0.5 M $NaClO_4$	1036	70.25 ± 0.09
10^{-2} M $HClO_4$ + 0.5 M $NaClO_4$	1036	70.19 ± 0.08
10^{-1} M $HClO_4$ + 0.5 M $NaClO_4$	1041	69.31 ± 0.07
1 M $HClO_4$	1057	67.22 ± 0.10

S3 Surface Characterization

Atomic force microscope (AFM) and scanning electron microscope (SEM) were employed to characterize the surface conditions of the new and used electrodes. Nanoscale topography images are acquired in tapping mode using an AFM (Bruker Icon) under ambient conditions with a humidity of approximately 48% (measured with TFA Digital Professional Thermo-Hygrometer KLIMA BEE). A heavily doped n-type Si cantilever with a resonance frequency of 85 kHz and a force constant of 2.7 N/m (SSS-FMR, Nanosensors) was used. An open-source software (Gwyddion) was utilized for post-processing the raw images and extracting statistical values.

Additionally, the surface morphology of an electrode was observed using a SEM (JSM-IT200 from Jeol Ltd.) under the high vacuum environment, with magnifications up to 10,000x. The aperture was positioned at a distance of 11.4 mm from the substrate. The beam voltage and probe current (PC) were set to 20 kV and 60 nA, respectively. The SEM images of the new and used electrodes are shown in Figure S2, with magnification increasing from left to right within each row. Among the scanned areas on the new electrode, no remarkable surface features were seen, neither for a large area (a), nor a smaller area (c).

Conversely, the damage on the electrode surface due the bubble detachment and surface cleaning between the experiments is evident at all magnification levels (d, e, f).

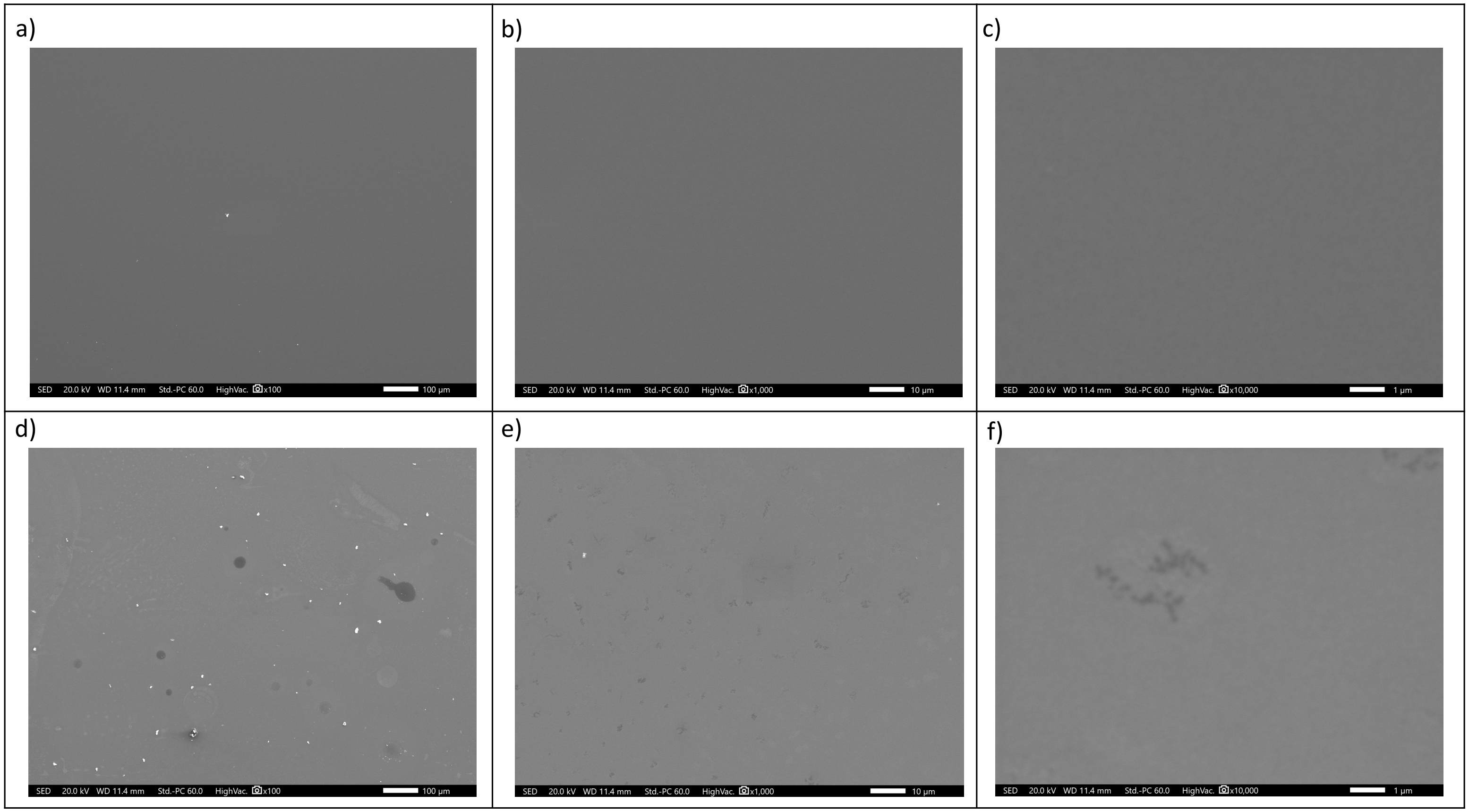

Figure S2: Scanning electron microscope (SEM) images of a new and used electrodes. Images (a), (b), and (c) represent the surface of the new electrode in three magnification levels (with scale bars of 100 µm, 10 µm, and 1 µm, respectively). On the other hand, images (d), (e), and (f) show the surface of a used electrode at the same scales.

S4 Wetting Characteristics

The wetting properties of the new and used electrodes were determined by sessile drop experiments. Similar to the pendant drop experiments, sessile drop experiments were also performed using an optical contact angle goniometer (OCA 15 Pro from Dataphysics Instruments), and a Hamilton syringe-needle pair. To determine the dynamic contact angles (θ_{adv} and θ_{rec}) and equilibrium contact angle (θ_{eq}) of the electrodes, two different techniques were employed. First, the dynamic contact angles of a new and a used electrode were measured using needle-in-drop sessile drop technique (see Figure 5(b,c) in the main text). In this technique, θ_{adv} is measured as the contact line spreads across the surface during the deposition of a drop. Right after that, the deposited amount is withdrawn by the syringe, and θ_{rec} is measured as the contact line shrinks. In the second technique, θ_{adv} and θ_{eq} of a drop were measured. θ_{adv} is found in the same way as the first technique. However, once the deposition is completed, the substrate is subjected to vibration to achieve the state in which the drop has the minimum Gibbs energy, i.e. the most stable equilibrium state.[6] Subsequently, θ_{eq} is determined (see Figure S3). Further details on this part can be found in a previous work.[7] The volume of each drop in both techniques was set to 10 µL as small amounts of drops affect the accuracy of the measurements.[8] The drops were pumped in and out at a constant pumping speed of 0.1 µL/s. The capillary numbers of a drop spreading over a used electrode typically fall within the range of $\mathcal{O}(10^{-7} - 10^{-8})$.

Figure 5 shows that the dynamic contact angles and the contact angle hysteresis, i.e. the difference between θ_{adv} and θ_{rec}. For a new electrode, the hysteresis remains around 20° across all electrolytes. However, it elevates to around 70° for a used electrode. This substantial change provides clear evidence that electrolysis experiments induce significant alterations in the electrode surface. Despite the pronounced differences in dynamic contact angles, θ_{eq} for both new and used electrodes are remarkably similar, as depicted in Figure S3.

Li et al.[9] reported a water contact angle of ≈ 81° for flat platinum film electrodes based

on sessile droplet experiments. Fernandez et al.[10] followed the water contact angle of electrolytically growing bubbles and found a value ≈ 68° for the case where a single bubble detaches and a new bubble forms. Hydrophobic and other surface contaminants are well-known to lead to finite contact angles on platinum, leading to the use of flame or strong acid treatment to obtain perfect wetting behaviour in the case of Wilhelmy plate measurements.[11,12] In our case, we measure the 'most stable' contact angle as an approximation of the equilibrium contact angle for our platinum electrode material and this value may be quite different compared to other Pt-electrodes depending on the nature of surface contaminants or pretreatments.

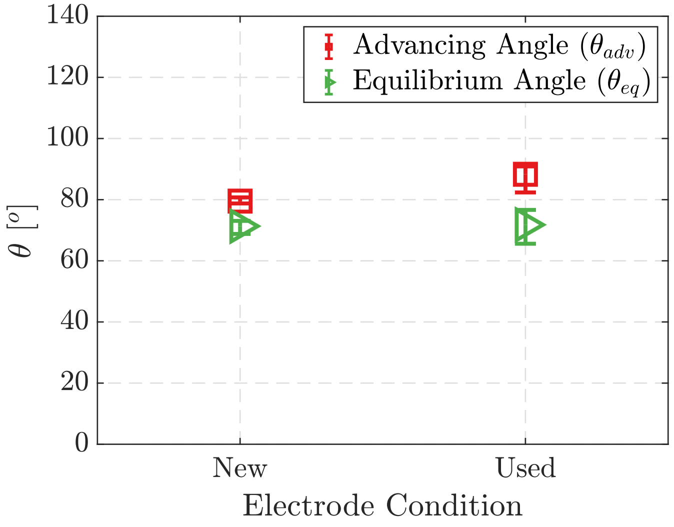

Figure S3: Advancing (θ_{adv}) and equilibrium (θ_{eq}) angles of water on a new and a used electrode, as determined through sessile drop experiments.

S5 Spreading Bubbles at Various Conditions

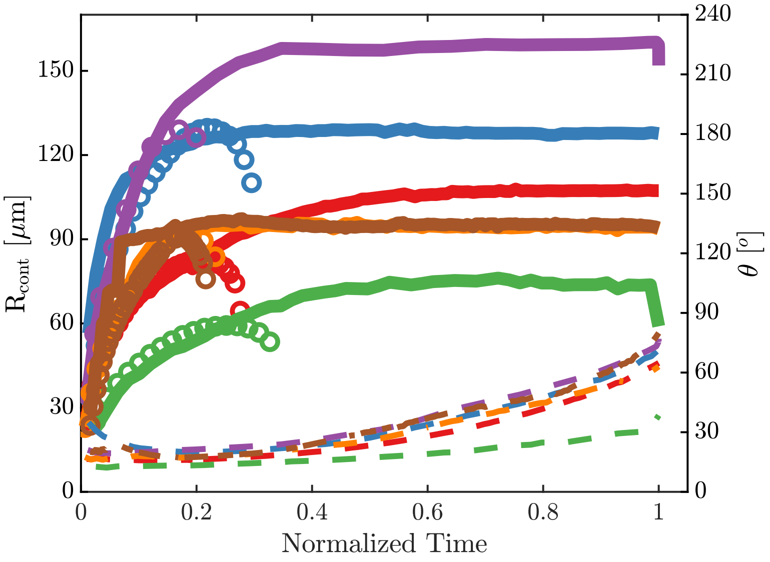

Bubble IDx	Electrode Material	Concentration [M]	Current Dens. [A/m²]
1	Pt	0.1	− 30
2	Pt	0.1	− 10
3	Pt	1	− 200
4	Pt	0.1	− 70
5	Pt	1	− 50
6	Ni	0.1	− 30

Figure S4: Contact radius (R_{cont}) and contact angle (θ) change in time for different bubbles at various experimental conditions.

References

(1) Meulenbroek, A.; Vreman, A.; Deen, N. Competing Marangoni effects form a stagnant cap on the interface of a hydrogen bubble attached to a microelectrode. *Electrochim. Acta* **2021**, *385*, 138298.

(2) Massing, J.; Mutschke, G.; Baczyzmalski, D.; Hossain, S. S.; Yang, X.; Eckert, K.; Cierpka, C. Thermocapillary convection during hydrogen evolution at microelectrodes. *Electrochim. Acta* **2019**, *297*, 929–940.

(3) Park, S.; Liu, L.; Demirkır, Ç.; van der Heijden, O.; Lohse, D.; Krug, D.; Koper, M. T. M. Solutal Marangoni effect determines bubble dynamics during electrocatalytic hydrogen evolution. *Nat. Chem.* **2023**, *15*, 1532–1540.

(4) Park, S.; Lohse, D.; Krug, D.; Koper, M. T. Electrolyte design for the manipulation of gas bubble detachment during hydrogen evolution reaction. *Electrochim. Acta* **2024**, *485*, 144084.

(5) Bashkatov, A.; Hossain, S. S.; Yang, X.; Mutschke, G.; Eckert, K. Oscillating Hydrogen Bubbles at Pt Microelectrodes. *Phys. Rev. Lett.* **2019**, *123*.

(6) Mittal, K. L. *Contact Angle, Wettability and Adhesion, Volume 6*; Taylor & Francis Group, 2009.

(7) Demirel, Ö. Metal organic frameworks at interfaces. Ph.D. thesis, University of Twente, Netherlands, 2020.

(8) Huhtamäki, T.; Tian, X.; Korhonen, J. T.; Ras, R. H. A. Surface-wetting characterization using contact-angle measurements. *Nat. Protoc.* **2018**, *13*, 1521–1538.

(9) Li, Y.; Zhang, H.; Xu, T.; Lu, Z.; Wu, X.; Wan, P.; Sun, X.; Jiang, L. Under-Water Superaerophobic Pine-Shaped Pt Nanoarray Electrode for Ultrahigh-Performance Hydrogen Evolution. *Adv. Funct. Mater.* **2015**, *25*, 1737–1744.

(10) Fernández, D.; Maurer, P.; Martine, M.; Coey, J. M. D.; Möbius, M. E. Bubble Formation at a Gas-Evolving Microelectrode. *Langmuir* **2014**, *30*, 13065–13074.

(11) Bewig, K. W.; Zisman, W. A. The Wetting of Gold and Platinum by Water. *J. Phys. Chem.* **1965**, *69*, 4238–4242.

(12) Momsen, W. E.; Smaby, J. M.; Brockman, H. L. The suitability of nichrome for measurement of gas-liquid interfacial tension by the wilhelmy method. *J. Colloid Interface Sci.* **1990**, *135*, 547–552.
